# Synthesis of High-Entropy Perovskite Hydroxides as Bifunctional Electrocatalysts for Oxygen Evolution Reaction and Oxygen Reduction Reaction

**DOI:** 10.3390/ma17122963

**Published:** 2024-06-17

**Authors:** Sangwoo Chae, Akihito Shio, Tomoya Kishida, Kosuke Furutono, Yumi Kojima, Gasidit Panomsuwan, Takahiro Ishizaki

**Affiliations:** 1 SIT Research Laboratories, Shibaura Institute of Technology, 3-7-5 Toyosu, Koto-ku, Tokyo 135-8548, Japan; chae@sic.shibaura-it.ac.jp; 2 Materials Science and Engineering, Graduate School of Engineering and Science, Shibaura Institute of Technology, 3-7-5 Toyosu, Koto-ku, Tokyo 135-8548, Japan; ac20014@shibaura-it.ac.jp (A.S.); mb23012@shibaura-it.ac.jp (T.K.); mb23035@shibaura-it.ac.jp (K.F.); mb23016@shibaura-it.ac.jp (Y.K.); 3 Department of Materials Engineering, Faculty of Engineering, Kasetsart University, Bangkok 10900, Thailand; gasidit.p@ku.ac.th; 4 College of Engineering, Shibaura Institute of Technology, 3-7-5 Toyosu, Koto-ku, Tokyo 135-8548, Japan

**Keywords:** high-entropy perovskite hydroxides, bifunctional electrocatalyst, oxygen reduction reaction, oxygen evolution reaction

## Abstract

Oxygen reduction reaction (ORR) and oxygen evolutionc reaction (OER) are important chemical reactions for a rechargeable lithium–oxygen battery (LOB). Recently, high-entropy alloys and oxides have attracted much attention because they showed good electrocatalytic performance for oxygen evolution reaction (OER) and/or oxygen reduction reaction (ORR). In this study, we aimed to synthesize and characterize CoSn(OH)_6_ and two types of high-entropy perovskite hydroxides, that is, (Co_0.2_Cu_0.2_Fe_0.2_Mn_0.2_Mg_0.2_)Sn(OH)_6_ (CCFMMSOH) and (Co_0.2_Cu_0.2_Fe_0.2_Mn_0.2_Ni_0.2_)Sn(OH)_6_ (CCFMNSOH). TEM observation and XRD measurements revealed that the high-entropy hydroxides CCFMMSOH and CCFMNSOH had cubic crystals with sides of approximately 150–200 nm and crystal structures similar to those of perovskite-type CSOH. LSV measurement results showed that the high-entropy hydroxides CCFMMSOH and CCFMNSOH showed bifunctional catalytic functions for the ORR and OER. CCFMNSOH showed better catalytic performance than CCFMMSOH.

## 1. Introduction

Lithium–oxygen batteries (LOBs) use metallic lithium for the anode and oxygen for the cathode. Thus, they theoretically have a high energy density [1,2]. The current driving range is 160 km when a mainstream lithium-ion battery is installed in an electric vehicle, but a driving range of 500 km can be achieved with an LOB [3]. LOBs have a structure in which a metallic lithium anode and cathode catalyst, such as porous carbon, are connected by an electrolyte. The chemical reaction formula for a LOB is shown in Equation (1) [4].

(1) $$2{\mathrm{Li}}^{+}+2e^{-}+{\;O}_{2}⇄{\mathrm{Li}}_{2}O_{2}$$

During the discharge reaction, lithium peroxide (Li_2_O_2_) is produced as a discharge product, with the reaction ending when the passive lithium peroxide covers the cathode [4]. During charging, the application of voltage decomposes the lithium peroxide and releases oxygen [4]. The occurrence of the electrochemical reaction shown in Equation (1) generates a potential of 2.96 V. Thus, the battery has extremely high energy. However, the potential during the actual charging and discharging reaction deviates from the theoretical potential, and a large overpotential is required to cause the chemical reaction shown in Equation (1). Therefore, catalyst materials to reduce this overpotential have currently been developed [4], with the goal of using a catalyst for an oxygen reduction reaction (ORR) in the cathode material to reduce the overpotential during discharging [4,5] and a catalyst for an oxygen evolution reaction (OER) in the cathode material to reduce the overpotential during charging [6,7].

Catalytic materials that exhibit high ORR activity include platinum and palladium, and catalyst materials that exhibit high OER activity include ruthenium oxide [8]. However, both have high costs because of the use of precious metals, with the additional problem of limited reserves. Therefore, alternative catalyst materials need to be developed. Transition metal oxides are inexpensive, available in large quantities, and have been reported to exhibit high catalytic activity as LOB cathode catalysts [9]. Cobalt-based oxides (Co_3_O_4_, CoO) in particular act as catalysts for both the ORR and OER [10] and have been reported to exhibit high catalytic activity as LOB cathode catalysts. CoSn(OH)_6_ is a tin-based metal hydroxide with a perovskite structure and exhibits catalytic activity for the ORR and OER [11]. Thus, the use of CoSn(OH)_6_ as a cathode catalyst material for LOBs is expected [12]. 

Recently, high-entropy alloys and oxides have attracted much attention because they have been reported to be good electrocatalytic materials as a result of their unique and adjustable structural characteristics [13,14]. Loffler et al. used combinatorial co-sputtering to fabricate transition metal-based high-entropy alloy nanoparticles and reported ORR activity exceeding that of Pt/C [13]. Following this report, high-entropy alloys have attracted attention as ORR catalyst materials [14]. High-entropy alloys are composed of five or more different elements and contain 5–35% of each element [14]. Their increased entropy has been shown to distort the crystal lattice and increase the number of active sites, thereby improving their catalytic activity. Therefore, active research has been conducted on increasing the entropy of metal oxides and metal nitrides [15]. Sarker et al. used the nebulized spray pyrolysis method to fabricate (Co_0.2_Cu_0.2_Mg_0.2_Ni_0.2_Zn_0.2_)O and found that it exhibited an excellent Li storage capacity as an anode material for lithium-ion batteries [16]. Based on these results, it can be expected that high-entropy metal hydroxides also exhibit excellent catalytic properties for the ORR and OER.

In this study, we aimed to synthesize and characterize CoSn(OH)_6_ and two types of high-entropy perovskite hydroxides: (Co_0.2_Cu_0.2_Fe_0.2_Mn_0.2_Mg_0.2_)Sn(OH)_6_ (CCFMMSOH) and (Co_0.2_Cu_0.2_Fe_0.2_Mn_0.2_Ni_0.2_)Sn(OH)_6_ (CCFMNSOH). The choice of these elements for sample synthesis is based on their potential to enhance catalytic activity due to their varied chemical properties and ability to form high-entropy compounds. Cobalt (Co) and nickel (Ni) are known for their catalytic activities in both ORR and OER, while copper (Cu), iron (Fe), manganese (Mn), and magnesium (Mg) contribute to the structural stability and overall catalytic performance of the high-entropy hydroxides. By incorporating these elements, we aimed to evaluate their bifunctional catalytic performances concerning ORR and OER, potentially advancing the development of cost-effective, high-performance LOBs.

## 2. Materials and Methods

### 2.1. Synthesis of High-Entropy Perovskite Hydroxides

1 mmol of cobalt(II) chloride (purity 97.0%, manufactured by Wako Pure Chemical Industries Ltd., Saitama, Japan) and 1 mmol of sodium citrate (purity 99.0%, manufactured by Sigma-Aldrich) were dissolved in 31 mL of ultrapure water (at a temperature of 25 °C, with a specific resistivity of 18.2 MΩ). Then, 1 mmol of tin(IV) chloride pentahydrate (purity 98%, manufactured by Kanto Chemical Co., Inc., Tokyo, Japan) was dissolved in 4 mL of ethanol (purity 99.5%, manufactured by Kanto Chemical Co., Inc., Tokyo, Japan). These solutions were mixed and stirred for 5 min. We then dripped 5 mL of an aqueous solution of sodium hydroxide (purity 97%, manufactured by Kanto Chemical Co., Inc., Tokyo, Japan), adjusted to 2 mol/L, into this mixture and stirred it for 1 h. The precipitate was then separated by suction filtration and dried overnight in an electric furnace at 80 °C to synthesize CoSn(OH)_6_ (CSOH).

Following the same procedure, 0.2 mmol each of cobalt(II) chloride, copper(II) chloride (purity 95%, manufactured by Kanto Chemical Co., Inc., Tokyo, Japan), iron(II) chloride tetrahydrate (purity 99.0%, manufactured by Wako Pure Chemical Industries Ltd., Saitama, Japan), manganese(II) chloride tetrahydrate (purity 99.0%, manufactured by Kanto Chemical Co., Inc. Tokyo, Japan), and magnesium(II) chloride tetrahydrate (purity 97.0%, manufactured by Wako Pure Chemical Industries Ltd., Saitama, Japan) was used in place of the 1 mmol of cobalt(II) chloride to synthesize (Co_0.2_Cu_0.2_Fe_0.2_Mn_0.2_Mg_0.2_)Sn(OH)_6_ (CCFMMSOH). A total of 0.2 mmol each of cobalt(II) chloride, copper(II) chloride, iron(II) chloride tetrahydrate, manganese(II) chloride tetrahydrate, and nickel(II) chloride (purity 98.0%, manufactured by Tokyo Chemical Industry Co., Ltd.) were used to synthesize (Co_0.2_Cu_0.2_Fe_0.2_Mn_0.2_Ni_0.2_)Sn(OH)_6_ (CCFMNSOH). These compounds were synthesized using the same procedure as for CSOH.

### 2.2. Evaluation of the Synthesized High-Entropy Perovskite Hydroxides

The crystal structure of each synthesized sample was identified using X-ray diffraction (XRD) (Smart Lab, manufactured by Rigaku Co., Ltd., Tokyo, Japan). The XRD measurements were conducted using CuKα rays with a measurement range of 2*θ* = 15°–60° and a scanning speed of 10.0°/min. The metal concentration of each synthesized sample was measured using an inductively coupled plasma optical emission spectrometer (5110, manufactured by Agilent Technologies Inc., Santa Clara, CA, USA; ICP-OES) and a calibration curve method. ICP mixed standard solution H (10 mg/L, manufactured by Kanto Chemical Co., Inc., Saitama, Japan) was used as the standard solution for quantitative analysis of the synthesized samples. A transmission electron microscope (JEM-2100, manufactured by JEOL Ltd., Tokyo, Japan; TEM) was used to observe the microstructure and analyze the composition of each sample. An X-ray photoelectron spectrometer (JPS-9010MC, manufactured by JEOL Ltd., Tokyo, Japan; XPS) was used to conduct qualitative analyses of the bonding states and substances. XPS measurements were conducted using Mg-Kα X-rays at a voltage of 10 kV and a current of 25 mA. The 284.5 eV peak of the C 1s spectrum was used for the charge-up correction of the obtained spectrum. A specific surface area/pore distribution measurement device (TriStar II 3020, manufactured by Micromeritics Co., Ltd., Norcross, GA, USA) was used to measure the specific surface area of each sample. At the time of measurement, a container was filled with liquid nitrogen, and measurements were taken using nitrogen gas. The sample was degassed at 100 °C for 24 h as a pretreatment.

### 2.3. Electrochemical Measurements

Each synthesized sample was used as the active material of an electrode and tested for catalytic performances for the ORR and OER using a dual electrochemical analyzer (ALS704ES, manufactured by BAS, Tokyo, Japan). Commercial Pt/C and RuO_2_ were also used as references for active materials for ORR and OER, respectively. The slurry was prepared to be applied to the electrode by placing 5 mg of the synthesized sample in a mixed solution of 480 µL of ultrapure water, 480 µL of ethanol, and 40 µL of Nafion (purity 5 wt%, manufactured by Sigma-Aldrich, Saint Louis, MO, USA), which was dispersed ultrasonically (20 °C, 40 kHz). A total of 7.5 µL of the prepared slurry was dripped onto a Pt (ring)-GC (disk) electrode (inner/outer-ring diameters: 5.0/7.0 mm, *A*_ring_ = 0.188 cm^2^, disk diameter: 4 mm, *A*_disk_ = 0.126 cm^2^) and it was dried overnight under atmospheric pressure (amount of active material fixed on electrode: 0.024 mg). This electrode was used as the working electrode, platinum as the counter electrode, and a silver/silver chloride (Ag/AgCl) electrode in a saturated aqueous solution of potassium chloride as the reference electrode. A 0.1 mol/L aqueous solution of potassium hydroxide (pH = 13) was used as the electrolyte. The dissolved oxygen was removed by flowing nitrogen gas into the solution for 20 min using a rotating ring-disk electrode device (RRDE-3A, manufactured by BAS, Tokyo, Japan), after which we purged the oxygen to create an oxygen-saturated environment for the electrolyte. The ORR and OER performances were measured using linear sweep voltammetry (LSV) at a rotation speed of 1500 rpm. The LSV measurements were conducted at a scan rate of 0.01 V/s and scan ranges for the ORR and OER of −1.0 to 0 V and 0 to 1.0 V, respectively, relative to the Ag/AgCl electrode potential. All the potentials described in this paper were converted to reversible hydrogen electrode (RHE) potentials using Equation (2) [17].

*E*_RHE_ = *E*_Ag/AgCl_ + 0.197 + 0.0591 × pH (2)

## 3. Results and Discussion

### 3.1. Characterization of the Synthesized High-Entropy Perovskite Hydroxides

**Figure 1:**
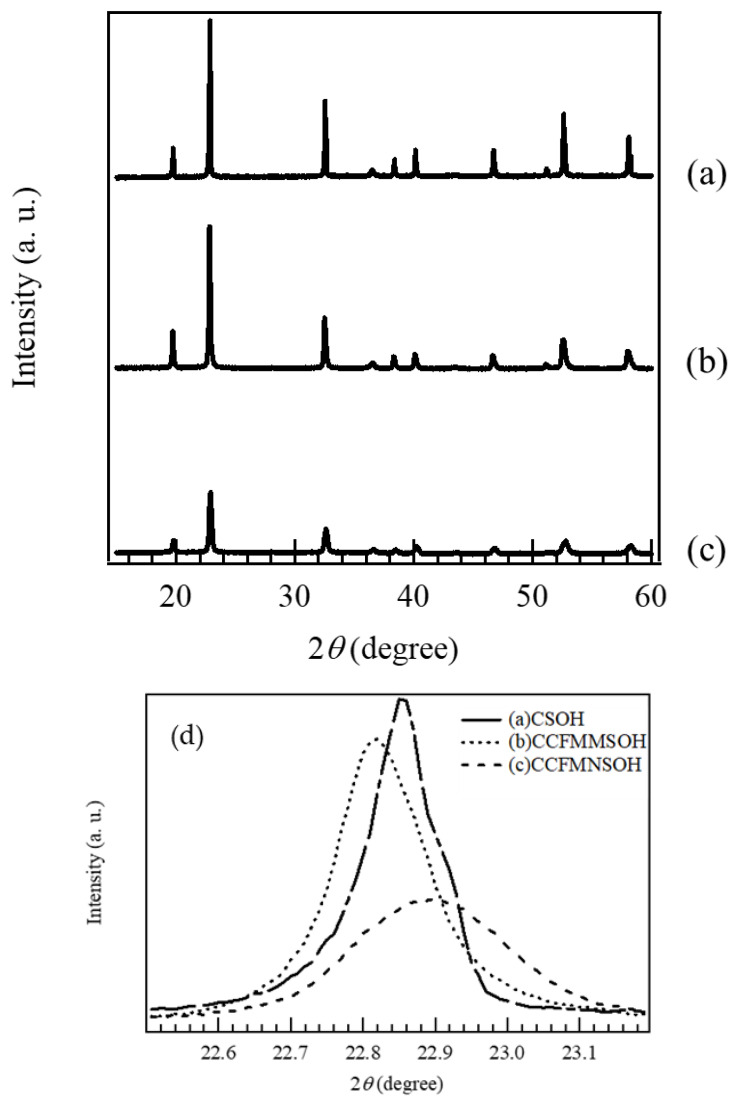
XRD patterns of (**a**) CSOH, (**b**) CCFMMSOH, and (**c**) CCFMNSOH, and (**d**) enlarged XRD patterns of CSOH, CCFMMSOH, and CCFMNSOH at 2*θ* = 22.5° to 23.2°.

Figure 1 shows the XRD patterns of the synthesized (a) CSOH, (b) CCFMMSOH, and (c) CCFMNSOH. The XRD pattern of CSOH shown in Figure 1a has diffraction peaks near 2*θ* = 20°, 23°, 32.5°, 36°, 38°, 40°, 47°, 51°, 53°, 58°, 62°, 68°, 73°, 78°, and 82°, corresponding to the 111, 200, 220, 310, 311, 322, 400, 331, 420, 422, 511, 440, 600, 620, and 622 reflections of CoSn(OH)_6_, respectively [12]. These results indicate the synthesis of CoSn(OH)_6_. Additionally, Figure 1b,c show diffraction peaks similar to those of CSOH present on the XRD patterns of CCFMMSOH and CCFMNSOH. We infer from these results that CCFMMSOH and CCFMNSOH have crystal structures similar to those of CSOH. Figure 1d shows an enlargement of the XRD pattern in the range of 2*θ* = 22.5°–23.2°. It can be seen from Figure 1d that the peak positions of the 200 reflections in the XRD patterns of CCFMMSOH and CCFMNSOH shifted relative to those for CSOH. This peak shift indicates a change in the lattice spacing of the (200) plane. We inferred that this was the result of distortion in the crystal lattice caused by replacing the Co site with an element species with a different atomic radius. 

Furthermore, the XRD peaks of CCFMMSOH and CCFMNSOH were broader than those of CSOH, which indicated a lower crystallinity in CCFMMSOH and CCFMNSOH than in CSOH. The peak of CCFMNSOH in particular broadened considerably, suggesting that its crystallinity decreased the most. Particularly, the peak broadening for CCFMNSOH is more pronounced, which implies a significant decrease in its crystallinity. This reduction in crystallinity could be attributed to the introduction of high-entropy elements, which may disrupt the regular crystal lattice and result in a more disordered structure. The variation in crystallinity among these compounds suggests a potential impact on their electrocatalytic performance, as crystallinity can influence the active surface area and the accessibility of active sites [18].

**Figure 2:**
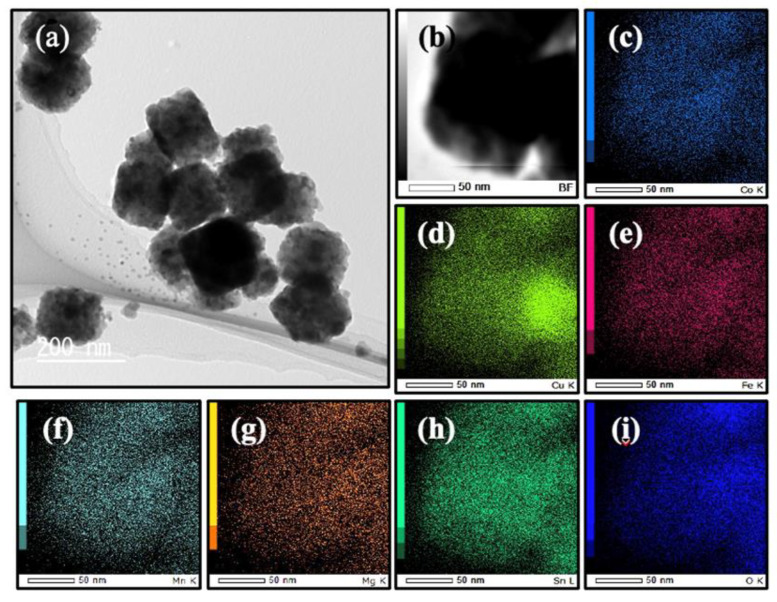
(**a**) TEM image, (**b**) STEM image, and elemental mapping images of CCFMMSOH: (**c**) Co, (**d**) Cu, (**e**) Fe, (**f**) Mn, (**g**) Mg, (**h**) Sn, and (**i**) O.XRD patterns of (**a**) Co-NC@CNT, (**b**) Ni-NC@CNT, and (**c**) CSCNT.

**Figure 3:**
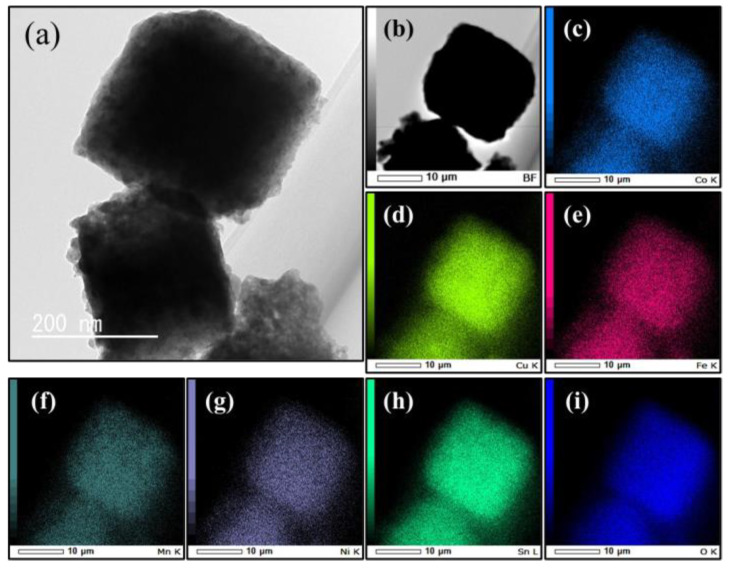
(**a**) TEM image, (**b**) STEM image, and elemental mapping images of CCFMNSOH: (**c**) Co, (**d**) Cu, (**e**) Fe, (**f**) Mn, (**g**) Ni, (**h**) Sn, and (**i**) O.

Table 1 summarizes the concentration ratios of the metal elements in CCFMMSOH and CCFMNSOH obtained from the ICP-OES measurement results. The results in Table 1 indicate that CCFMMSOH and CCFMNSOH contained metal components other than tin at a ratio of approximately 20 at%. It is predicted from these element ratios that the synthesized CCFMMSOH and CCFMNSOH have high entropy states. Figure 2 and Figure 3 show TEM and elemental mapping images of CCFMMSOH and CCFMNSOH, respectively. The TEM images in Figure 2a and Figure 3a show that both CCFMMSOH and CCFMNSOH were cubic crystals with sides of approximately 150–200 nm. Additionally, the STEM-EDS elemental mapping images in Figure 2c–i and Figure 3c–i show that the constituent elements of CCFMMSOH and CCFMNSOH were uniformly dispersed within the sample without agglomeration. The results for the elemental concentration ratios found using the XRD, ICP-OES, TEM images, and elemental mapping images by STEM-EDS showed that CCFMMSOH and CCFMNSOH were crystals with crystal structures similar to those of CSOH, the ratio of introduced elements was within the definition of high-entropy materials [14], and the introduced elements were evenly distributed within the sample. These aspects suggested that all the synthesized substances were high-entropy metal hydroxides.

**Figure 4:**
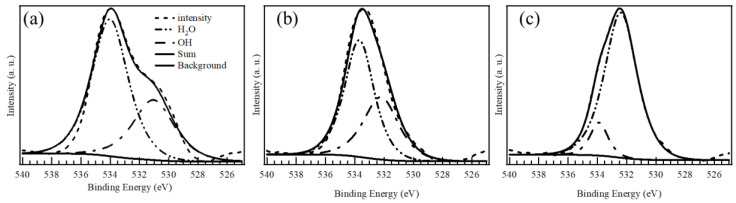
High-resolution XPS O1s spectra and deconvolution of (**a**) CSOH, (**b**) CCFMMSOH, and (**c**) CCFMNSOH.

Figure 4 shows the XPS O 1*s* spectra of (a) CSOH, (b) CCFMMSOH, and (c) CCFMNSOH. We can confirm from the XPS spectra in Figure 4 that there is one broad peak in the O 1*s* spectra of CSOH, CCFMMSOH, and CCFMNSOH, and that this peak can be deconvoluted into two waveforms, with one due to the OH bond around 532–533 eV and the other due to the H_2_O bond around 533–534 eV [19]. Table 2 lists the relative ratios of the OH bonds and H_2_O-derived bonds in CSOH, CCFMMSOH, and CCFMNSOH as determined from the waveform separation of the O 1s spectra. OH has been reported to originate from water molecules adsorbed to oxygen vacancies, and H_2_O has been reported to originate from water molecules irreversibly adsorbed to the surface [19]. The high-entropy material CCFMNSOH had an increased ratio of OH bond species, which was inferred to be due to an increase in oxygen vacancies. This result was in agreement with the XRD result that the crystallinity of CCFMNSOH decreased the most. This decrease in crystallinity could further facilitate the formation of oxygen vacancies, thereby enhancing the material’s surface reactivity. The correlation between the increased OH bond ratio and the presence of oxygen vacancies in CCFMNSOH highlights the material’s unique surface properties, which are critical to its catalytic performance [20].

**Figure 5:**
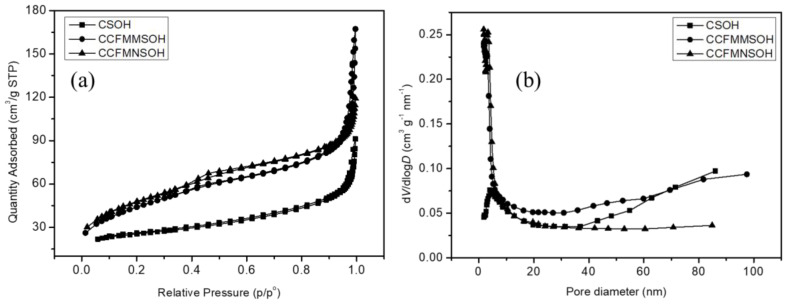
(**a**) N_2_ adsorption-desorption plots and (**b**) pore diameter distribution curves of CSOH, CCFMMSOH, and CCFMNSOH.

Figure 5 shows the N_2_ adsorption-desorption isotherms for CSOH, CCFMMSOH, and CCFMNSOH. The isotherm curves show a characteristic Type IV profile with an H1 hysteresis loop, in line with the International Union of Pure and Applied Chemistry (IUPAC) nomenclature, indicating the presence of a mesoporous structure for all three materials. This mesoporous structure is crucial as it relates to the number of active sites available for catalytic reactions, suggesting that materials with larger surface areas can offer higher catalytic activities. The pore size distribution, determined through the Barrett-Joyner-Halenda (BJH) method, revealed sharp peaks at 4.62 nm for CCFMNSOH, 4.27 nm for CCFMMSOH, and 3.65 nm for CSOH. These results imply that the pore sizes within these materials are well-defined and contribute to their catalytic properties. The Brunauer–Emmett–Teller (BET)-specific surface area measurements for each material are listed in Table 3. CCFMNSOH demonstrated the highest surface area at 168.33 m^2^/g, followed by CCFMMSOH at 158.72 m^2^/g, and CSOH at 84.67 m^2^/g. This descending order of surface areas aligns with the expectation that a higher surface area, hence a larger number of accessible active sites, correlates with an increase in catalytic activity [21].

### 3.2. Electrocatalytic Activity of the Synthesized High-Entropy Perovskite Hydroxides

**Figure 6:**
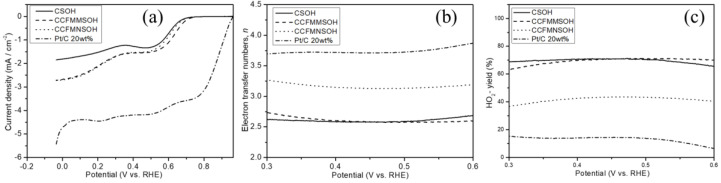
(**a**) ORR curves of CSOH, CCFMMSOH, CCFMNSOH, and Pt/C 20 wt% in O_2_-saturated 0.1 M KOH aqueous solution. Change in (**b**) electron transfer numbers (*n*), and (**c**) HO_2_^−^ yields of CSOH, CCFMMSOH, CCFMNSOH, and Pt/C 20 wt.% as a function of potential.

Figure 6a shows the LSV curves obtained using CSOH, CCFMMSOH, CCFMNSOH, and Pt/C 20 wt.% as active electrode materials. An increase in current density was observed, which is indicative of enhanced catalytic performance for ORR on the LSV curves for CSOH, CCFMMSOH, and CCFMNSOH, as shown in Figure 6a. The onset potentials for ORR of CSOH, CCFMMSOH, CCFMNSOH, and Pt/C 20 wt.% were identified to be 0.66, 0.71, 0.69, and 0.94 V, respectively, while their half-wave potentials (E_1/2_) were 0.586, 0.635, 0.607, and 0.830 V, respectively. Notably, CCFMMSOH exhibited the highest onset and half-wave potential values among all synthesized samples. A sample with a higher onset or half-wave potential is generally considered to possess superior catalytic capability, attributed to the lower energy barrier for electron transfer required for the ORR. The current densities at 0.5 V for CSOH, CCFMMSOH, CCFMNSOH, and Pt/C 20 wt.% were measured to be −0.79, −1.54, −1.52, and −4.39 mA/cm^2^, respectively. These results indicate that CCFMMSOH demonstrates the highest current density value among all synthesized materials. Figure 6b,c elucidates the variations in electron transfer number (*n*) and hydrogen peroxide (HO_2_^−^) production rate as a function of potential for all synthesized samples and Pt/C 20 wt.%. The *n* values for CSOH, CCFMMSOH, CCFMNSOH, and Pt/C 20 wt.% were approximately estimated to be 2.73, 2.28, 3.33, and 3.71, respectively, as shown in Table 4. The formation rate of HO_2_^−^ for CSOH, CCFMMSOH, CCFMNSOH, and Pt/C 20 wt.% was increased with a decrease in the *n* values. For CCFMNSOH, the *n* value was approximately 3.33, suggesting that ORR proceeds through a pathway involving both two- and four-electron transfers. Moreover, the *n* value for CCFMNSOH remained relatively stable across a broad potential range, indicating a smoother and more electrochemically stable ORR process. In the case of Pt/C, an *n* value of approximately 3.71 resulted in a relatively low HO_2_^−^ yield of about 14.40%, confirming that ORR predominantly occurs via the four-electron pathway. These outcomes confirm that high-entropy hydroxide exhibits superior catalytic activity compared to CSOH. 

**Figure 7:**
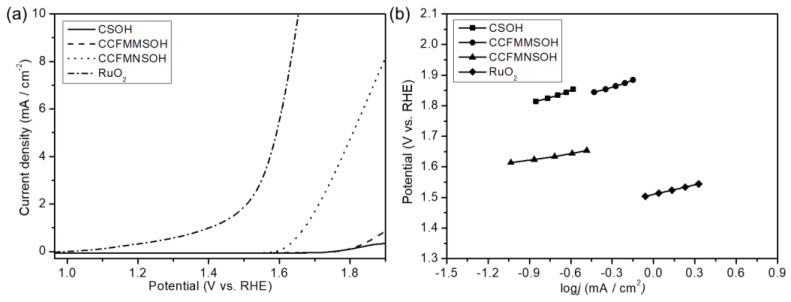
(**a**) OER curves of CSOH, CCFMMSOH, CCFMNSOH, and RuO_2_ in O_2_-saturated 0.1 M KOH aqueous solution. (**b**) Tafel plots of the composite samples synthesized at CSOH, CCFMMSOH, CCFMNSOH, and commercial RuO_2_.

Figure 7a shows the LSV curves for CSOH, CCFMMSOH, CCFMNSOH, and RuO_2_ used as active electrode materials. An increase in current density related to the OER was observed on the LSV curves for CSOH, CCFMMSOH, CCFMNSOH, and RuO_2_. The onset potentials of CSOH, CCFMMSOH, CCFMNSOH, and RuO_2_ for the OER were found to be 1.77, 1.77, 1.63, and 1.53 V vs. RHE, respectively. Narahara et al. reported that the onset potentials of the CSOH synthesized by the solution plasma process (SPP) were found to be in the range of 1.48 to 1.558 V vs. RHE [12]. The onset potential of our CSOH for OER was higher than that of CSOHs synthesized by SPP, indicating a higher overpotential. The potentials required to reach 5 mA/cm^2^ for CCFMNSOH and RuO_2_ are estimated to be 1.80 and 1.60 V vs. RHE, respectively, whereas the current density values of CSOH and CCFMMSOH were not achieved at 5 mA/cm^2^. These findings suggest that although the synthesized CSOH and CCFMMSOH exhibited catalytic activity for OER, their performance was not so high. The CCFMNSOH demonstrated the lowest onset potential and the highest current density for the OER, indicating the most superior catalytic performance among the synthesized samples. Figure 7b presents Tafel plots after OER onset for all synthesized samples and commercial RuO_2_. The Tafel slopes for CSOH, CCFMMSOH, CCFMNSOH, and RuO_2_ were found to be 251, 105, 69, and 71 mV/dec., respectively. It was reported that the Tafel slope of the CSOH synthesized by SPP was found to be 69.58 to 82.76 mV/dec [12]. From the comparison of our CSOH with CSOH synthesized by SPP for the onset potential and Tafel slope values, it was found that the electrocatalytic performance of our CSOH for OER was lower than that of CSOH synthesized by SPP. This difference in the electrocatalytic performance of OER could be due to the difference in the structural and electrical properties of the synthesized CSOH. 

The CCFMNSOH had the lowest Tafel slope value among all synthesized samples in this study and was lower than commercial RuO_2_. It is hypothesized that the difference in oxygen vacancies on the surface, which have been reported to serve as active sites for the OER [22], could produce the observed differences in catalytic activity. The XPS results indicated a higher relative ratio of OH bonds due to oxygen vacancies on the surface of CCFMNSOH compared to CCFMMSOH, suggesting enhanced catalytic performance of CCFMNSOH for the OER. This study confirmed the occurrence of both ORR and OER across all samples, indicating that CCFMMSOH and CCFMNSOH can function as bifunctional catalysts. Additionally, XRD patterns revealed a shift in the peak of the (200) plane of the high-entropy compound, suggesting a change in the lattice constant. Alterations in the electronic state due to the change in the lattice constant may have a relationship with OER activity [23]. A larger surface area has been associated with higher reactivity in catalytic reactions [24]. Our study further supports this correlation, highlighting the influence of specific surface areas on OER activity. 

The results of the ICP and the LSV measurements revealed that the small compositional changes (Co, Cu, Fe, Mn, Mg, and Ni) in the synthesized samples, i.e., the incorporation of different elements at the Co site in the high-entropy perovskite hydroxides, could affect the electrocatalytic activity for OER and ORR. For instance, the addition of Ni to CCFMNSOH resulted in a higher concentration of oxygen vacancies and an increased surface area, both of which affected the catalytic performance [25]. Similarly, the specific combination of Co, Cu, Fe, Mn, and Mg in CCFMMSOH provided a unique surface reactivity that improved ORR activity [26]. The incorporation of different elements at the Co site in the high-entropy perovskite hydroxides significantly influenced the catalytic activities for both ORR and OER. The CCFMNSOH exhibited the highest electrocatalytic performance among all synthesized samples in this study due to its increased oxygen vacancies and surface area, along with the beneficial effects of nickel. These findings highlight the importance of compositional tuning in the development of high-performance catalysts for energy storage applications.

## 4. Conclusions

The high-entropy hydroxides CCFMMSOH and CCFMNSOH, which had crystal structures similar to those of perovskite-type CSOH, were successfully synthesized by the coprecipitation method at room temperature and atmospheric pressure. However, their structures had oxygen defects, as revealed by the number of OH bonds caused by oxygen defects compared to CSOH. LSV measurement results showed that the high-entropy hydroxides CCFMMSOH and CCFMNSOH had bifunctional catalytic functions for the ORR and OER. CCFMNSOH was also shown to have better catalytic performance than CCFMMSOH. It was found that the catalytic performance improved with an increase in the relative ratio of the OH bond species as a result of an increase in the oxygen defects on the surface of the high-entropy hydroxides, as well as their surface areas. These findings underscore the potential for developing novel catalytic materials utilizing high-entropy hydroxides. Notably, the capacity to enhance catalytic performance through the manipulation of oxygen defects opens up promising avenues for applications in energy conversion and storage technologies. Moreover, further exploration into the relationship between the structural characteristics of these high-entropy hydroxides and their catalytic efficiency could significantly advance our understanding in this field and lead to the creation of more effective catalytic materials. Consequently, future investigations should include a broader evaluation of the catalytic performance of high-entropy hydroxides with varied compositional elements and oxygen defect levels.

## Figures and Tables

**Table 1:** ICP-OES measurement results for CCFMMSOH and CCFMNSOH.

	Atomic Composition, at%					
	Co	Cu	Fe	Mn	Mg	Ni
CCFMMSOH	17.9	20.9	22.6	20.6	18.0	―
CCFMNSOH	18.8	19.5	21.8	20.2	―	19.7

**Table 2:** XPS measurement results for CSOH, CCFMMSOH, and CCFMNSOH.

		Relative Component Ratio, %		
Bonding	Peak Position (eV)	CSOH	CCFMMSOH	CCFMNSOH
OH	531–532	33.2	38.9	88.9
H_2_O	533–534	66.8	61.1	11.1

**Table 3:** The BET surface areas and pore diameters of various samples.

	Surface Area, m^2^/g	Pore Diameter (nm)
CSOH	84.67	4.62
CCFMMSOH	158.72	4.27
CCFMNSOH	169.33	3.65

**Table 4:** ORR catalyst performance of each sample.

Sample	ORR Onset Potential (V)	Electron Transfer-Numbers (*n*)	Formation Rate of HO_2_^−^ (%)	Half-Wave Potential (V)	Limiting Current Density (mA/cm^2^)
CSOH	0.660	2.73	63.40	0.586	−0.79
CCFMMSOH	0.710	2.58	71.13	0.635	−1.54
CCFMNSOH	0.690	3.13	43.13	0.607	−1.52
Pt/C 20 wt%	0.940	3.71	14.40	0.830	−4.39

## Data Availability

Data are contained within the article.
